# 2,2′-(3,3′-Dihexyl-2,2′-bithio­phene-5,5′-di­yl)bis­(4,4,5,5-tetra­methyl-1,3,2-dioxaborolane)

**DOI:** 10.1107/S1600536811050938

**Published:** 2011-11-30

**Authors:** Lin Huang, Huisheng Li

**Affiliations:** a Department of Chemistry and Biology, Xiangfan University, Xiangfan 441053, People’s Republic of China

## Abstract

In the title mol­ecule, C_32_H_52_B_2_O_4_S_2_, the two thio­phene rings are twisted by 67.34 (2)°. In the crystal, weak C—H⋯O hydrogen bonds link mol­ecules related by translation along the *a* axis into chains.

## Related literature

For potential applications of the title compound, see: Navarro *et al.* (2004 ▶); Usta *et al.* (2006 ▶); Buszek & Brown (2007 ▶); Montes *et al.* (2007 ▶). For related structures, see: Decken *et al.* (2008 ▶); Kleeberg *et al.* (2009 ▶).
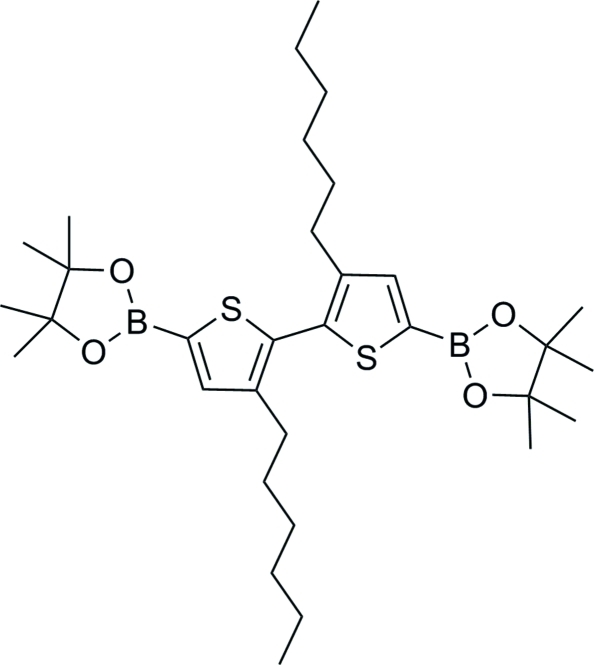


## Experimental

### 

#### Crystal data

C_32_H_52_B_2_O_4_S_2_
*M* *_r_* = 586.48
Monoclinic, 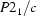

*a* = 11.5004 (11) Å
*b* = 13.6992 (13) Å
*c* = 21.300 (2) Å
β = 91.065 (2)°
*V* = 3355.1 (6) Å^3^
*Z* = 4
Mo *K*α radiation
μ = 0.19 mm^−1^
*T* = 100 K
0.16 × 0.12 × 0.10 mm

#### Data collection

Bruker APEX CCD diffractometer
Absorption correction: multi-scan (*SADABS*; Sheldrick, 1996 ▶) *T*_min_ = 0.970, *T*_max_ = 0.981
29998 measured reflections
9743 independent reflections
8009 reflections with *I* > 2σ(*I*)
*R*_int_ = 0.026

#### Refinement

*R*[*F* ^2^ > 2σ(*F* ^2^)] = 0.039
*wR*(*F* ^2^) = 0.118
*S* = 1.05
9743 reflections
371 parameters
H-atom parameters constrained
Δρ_max_ = 0.56 e Å^−3^
Δρ_min_ = −0.27 e Å^−3^

### 

Data collection: *SMART* (Bruker, 2001 ▶); cell refinement: *SAINT* (Bruker, 1999 ▶); data reduction: *SAINT*; program(s) used to solve structure: *SHELXS97* (Sheldrick, 2008 ▶); program(s) used to refine structure: *SHELXL97* (Sheldrick, 2008 ▶); molecular graphics: *SHELXTL* (Sheldrick, 2008 ▶); software used to prepare material for publication: *SHELXTL*.

## Supplementary Material

Crystal structure: contains datablock(s) I, global. DOI: 10.1107/S1600536811050938/cv5202sup1.cif

Structure factors: contains datablock(s) I. DOI: 10.1107/S1600536811050938/cv5202Isup2.hkl

Additional supplementary materials:  crystallographic information; 3D view; checkCIF report

## Figures and Tables

**Table 1:** Hydrogen-bond geometry (Å, °)

*D*—H⋯*A*	*D*—H	H⋯*A*	*D*⋯*A*	*D*—H⋯*A*
C30—H30*C*⋯O1^i^	0.98	2.53	3.2984 (18)	136

Symmetry code: (i) 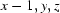.

**Fig. 1.**
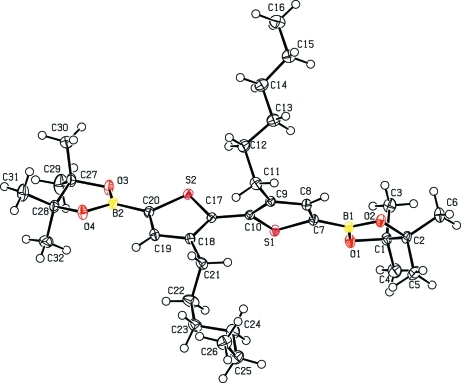
The molecular structure of (I) with the atom-numbering scheme. The displacement ellipsoids are drawn at the 30% probability level.

## supplementary crystallographic information

### Comment

Arylboronic acid and their esters are important reactants in the Suzuki-Miyaura cross-coupling reactions (Navarro *et al.*, 2004; Buszek *et al.*, 2007). Lots of functional organic compounds which have broad applications in material chemistry are prepared *via* the named reaction (Montes *et al.*, 2007; Usta *et al.*, 2006). We herein report the crystal structure of the title compound (I).

In (I) (Fig. 1), the geometric parameters of 2-(thiophene-2-yl)-4,4,5,5-tetramethyl- 1,3,2-dioxaborolane fragments are normal and comparable with those observed in related structures (Decken *et al.*, 2008; Kleeberg *et al.*, 2009). Two thiophene rings are twisted at 67.34 (2)°. In the crystal structure, weak intermolecular C—H···O hydrogen bonds (Table 1) link molecules related by translation along axis *a* into chains.

### Experimental

The powder form of the title compound (I) was purchased from Aldrich chemical company. Crystals of (I) suitable for X-ray data collection were obtained by slow evaporation of a acetone and MeOH solution in a ratio of 1:2 at room temperature for several days.

### Refinement

All H atoms were positioned geometrically (C—H = 0.93–0.97 Å) and refined as riding, allowing for free rotation of the methyl groups. The constraint *U*_iso_(H) = 1.2*U*_eq_(C) or 1.5*U*_eq_ (methyl C) was applied.

### Crystal data

**Table:**

C_32_H_52_B_2_O_4_S_2_	*F*(000) = 1272
*M**_r_* = 586.48	*D*_x_ = 1.161 Mg m^−^^3^
Monoclinic, *P*2_1_/*c*	Mo *K*α radiation, λ = 0.71073 Å
Hall symbol: -P 2ybc	Cell parameters from 9936 reflections
*a* = 11.5004 (11) Å	θ = 2.3–31.9°
*b* = 13.6992 (13) Å	µ = 0.19 mm^−^^1^
*c* = 21.300 (2) Å	*T* = 100 K
β = 91.065 (2)°	Block, yellow
*V* = 3355.1 (6) Å^3^	0.16 × 0.12 × 0.10 mm
*Z* = 4	

### Data collection

**Table:**

Bruker APEX CCD diffractometer	9743 independent reflections
Radiation source: fine-focus sealed tube	8009 reflections with *I* > 2σ(*I*)
graphite	*R*_int_ = 0.026
φ and ω scans	θ_max_ = 30.0°, θ_min_ = 1.8°
Absorption correction: multi-scan (*SADABS*; Sheldrick, 1996)	*h* = −16→9
*T*_min_ = 0.970, *T*_max_ = 0.981	*k* = −19→19
29998 measured reflections	*l* = −29→29

### Refinement

**Table:**

Refinement on *F*^2^	Primary atom site location: structure-invariant direct methods
Least-squares matrix: full	Secondary atom site location: difference Fourier map
*R*[*F*^2^ > 2σ(*F*^2^)] = 0.039	Hydrogen site location: inferred from neighbouring sites
*wR*(*F*^2^) = 0.118	H-atom parameters constrained
*S* = 1.05	*w* = 1/[σ^2^(*F*_o_^2^) + (0.0667*P*)^2^ + 1.0083*P*] where *P* = (*F*_o_^2^ + 2*F*_c_^2^)/3
9743 reflections	(Δ/σ)_max_ = 0.002
371 parameters	Δρ_max_ = 0.56 e Å^−^^3^
0 restraints	Δρ_min_ = −0.27 e Å^−^^3^

### Special details

**Table:**

Geometry. All e.s.d.'s (except the e.s.d. in the dihedral angle between two l.s. planes) are estimated using the full covariance matrix. The cell e.s.d.'s are taken into account individually in the estimation of e.s.d.'s in distances, angles and torsion angles; correlations between e.s.d.'s in cell parameters are only used when they are defined by crystal symmetry. An approximate (isotropic) treatment of cell e.s.d.'s is used for estimating e.s.d.'s involving l.s. planes.
Refinement. Refinement of *F*^2^ against ALL reflections. The weighted *R*-factor *wR* and goodness of fit *S* are based on *F*^2^, conventional *R*-factors *R* are based on *F*, with *F* set to zero for negative *F*^2^. The threshold expression of *F*^2^ > σ(*F*^2^) is used only for calculating *R*-factors(gt) *etc*. and is not relevant to the choice of reflections for refinement. *R*-factors based on *F*^2^ are statistically about twice as large as those based on *F*, and *R*- factors based on ALL data will be even larger.

### Fractional atomic coordinates and isotropic or equivalent isotropic displacement parameters (Å^2^)

**Table:**

	*x*	*y*	*z*	*U*_iso_*/*U*_eq_	
B1	1.10475 (12)	0.42742 (10)	0.66320 (6)	0.0165 (2)	
B2	0.42238 (12)	0.75653 (10)	0.66220 (6)	0.0177 (2)	
C1	1.31526 (14)	0.45484 (11)	0.78413 (7)	0.0302 (3)	
H1A	1.3426	0.5109	0.7599	0.045*	
H1B	1.3818	0.4142	0.7968	0.045*	
H1C	1.2756	0.4781	0.8216	0.045*	
C2	1.23132 (11)	0.39499 (9)	0.74398 (6)	0.0191 (2)	
C3	1.17597 (13)	0.31513 (11)	0.78332 (6)	0.0252 (3)	
H3A	1.1323	0.3449	0.8174	0.038*	
H3B	1.2369	0.2728	0.8011	0.038*	
H3C	1.1231	0.2763	0.7568	0.038*	
C4	1.37052 (12)	0.42478 (12)	0.65253 (7)	0.0292 (3)	
H4A	1.3879	0.4033	0.6098	0.044*	
H4B	1.4418	0.4234	0.6784	0.044*	
H4C	1.3396	0.4914	0.6512	0.044*	
C5	1.28072 (11)	0.35677 (9)	0.68070 (6)	0.0181 (2)	
C6	1.32667 (12)	0.25288 (10)	0.68271 (6)	0.0245 (3)	
H6A	1.2653	0.2088	0.6968	0.037*	
H6B	1.3933	0.2492	0.7120	0.037*	
H6C	1.3511	0.2335	0.6407	0.037*	
C7	0.99473 (10)	0.46694 (8)	0.62913 (5)	0.0160 (2)	
C8	0.94547 (11)	0.44202 (9)	0.57185 (6)	0.0167 (2)	
H8	0.9786	0.3946	0.5450	0.020*	
C9	0.84128 (10)	0.49275 (8)	0.55611 (5)	0.0152 (2)	
C10	0.81274 (10)	0.55846 (8)	0.60262 (5)	0.0140 (2)	
C11	0.76632 (12)	0.47441 (9)	0.49844 (6)	0.0205 (2)	
H11A	0.8136	0.4423	0.4660	0.025*	
H11B	0.7386	0.5376	0.4813	0.025*	
C12	0.66060 (12)	0.40942 (10)	0.51308 (7)	0.0245 (3)	
H12A	0.6217	0.4358	0.5505	0.029*	
H12B	0.6045	0.4129	0.4773	0.029*	
C13	0.69119 (12)	0.30299 (10)	0.52511 (7)	0.0255 (3)	
H13A	0.7580	0.3000	0.5550	0.031*	
H13B	0.7159	0.2730	0.4852	0.031*	
C14	0.59126 (13)	0.24350 (10)	0.55138 (7)	0.0266 (3)	
H14A	0.5805	0.2622	0.5958	0.032*	
H14B	0.5189	0.2604	0.5279	0.032*	
C15	0.60942 (14)	0.13357 (11)	0.54790 (8)	0.0321 (3)	
H15A	0.6157	0.1142	0.5033	0.038*	
H15B	0.6838	0.1169	0.5694	0.038*	
C16	0.51228 (16)	0.07546 (12)	0.57741 (9)	0.0380 (4)	
H16A	0.5080	0.0918	0.6221	0.057*	
H16B	0.5279	0.0055	0.5728	0.057*	
H16C	0.4382	0.0915	0.5564	0.057*	
C17	0.71460 (10)	0.62709 (8)	0.60397 (5)	0.0142 (2)	
C18	0.69640 (10)	0.70849 (8)	0.56718 (5)	0.0159 (2)	
C19	0.59343 (11)	0.75836 (9)	0.58463 (6)	0.0175 (2)	
H19	0.5672	0.8160	0.5640	0.021*	
C20	0.53489 (10)	0.71694 (9)	0.63341 (6)	0.0173 (2)	
C21	0.77521 (12)	0.74408 (9)	0.51653 (6)	0.0201 (2)	
H21A	0.7327	0.7420	0.4757	0.024*	
H21B	0.8428	0.6996	0.5138	0.024*	
C22	0.81873 (15)	0.84760 (10)	0.52837 (7)	0.0290 (3)	
H22A	0.8755	0.8465	0.5639	0.035*	
H22B	0.7523	0.8888	0.5409	0.035*	
C23	0.87603 (13)	0.89414 (10)	0.47171 (7)	0.0256 (3)	
H23A	0.8210	0.8912	0.4355	0.031*	
H23B	0.8911	0.9638	0.4811	0.031*	
C24	0.98960 (13)	0.84615 (11)	0.45327 (7)	0.0285 (3)	
H24A	0.9740	0.7773	0.4417	0.034*	
H24B	1.0435	0.8461	0.4901	0.034*	
C25	1.04875 (13)	0.89695 (11)	0.39843 (7)	0.0277 (3)	
H25A	1.0532	0.9679	0.4071	0.033*	
H25B	1.1293	0.8721	0.3953	0.033*	
C26	0.98547 (14)	0.88121 (11)	0.33606 (7)	0.0297 (3)	
H26A	0.9815	0.8112	0.3269	0.045*	
H26B	1.0275	0.9145	0.3027	0.045*	
H26C	0.9065	0.9078	0.3383	0.045*	
C27	0.26538 (12)	0.77376 (10)	0.72424 (7)	0.0228 (3)	
C28	0.26948 (11)	0.85917 (9)	0.67599 (6)	0.0197 (2)	
C29	0.24609 (16)	0.80293 (14)	0.79156 (8)	0.0398 (4)	
H29A	0.2499	0.7449	0.8184	0.060*	
H29B	0.1694	0.8335	0.7950	0.060*	
H29C	0.3064	0.8494	0.8050	0.060*	
C30	0.17912 (14)	0.69466 (11)	0.70388 (10)	0.0402 (4)	
H30A	0.1920	0.6774	0.6599	0.060*	
H30B	0.0996	0.7190	0.7084	0.060*	
H30C	0.1904	0.6367	0.7303	0.060*	
C31	0.15711 (14)	0.87827 (12)	0.64025 (8)	0.0343 (3)	
H31A	0.1682	0.9321	0.6107	0.051*	
H31B	0.0962	0.8956	0.6698	0.051*	
H31C	0.1340	0.8194	0.6170	0.051*	
C32	0.31596 (14)	0.95370 (11)	0.70410 (8)	0.0326 (3)	
H32A	0.3881	0.9404	0.7278	0.049*	
H32B	0.2582	0.9814	0.7323	0.049*	
H32C	0.3316	1.0002	0.6703	0.049*	
O1	1.13638 (8)	0.45839 (7)	0.72232 (4)	0.02101 (19)	
O2	1.17824 (8)	0.35991 (7)	0.63862 (4)	0.01828 (17)	
O3	0.38074 (8)	0.72931 (7)	0.71924 (5)	0.0244 (2)	
O4	0.35654 (8)	0.82553 (7)	0.63193 (4)	0.02210 (19)	
S1	0.91171 (3)	0.55548 (2)	0.664168 (13)	0.01627 (7)	
S2	0.60692 (3)	0.61359 (2)	0.658712 (14)	0.01632 (7)	

### Atomic displacement parameters (Å^2^)

**Table:**

	*U*^11^	*U*^22^	*U*^33^	*U*^12^	*U*^13^	*U*^23^
B1	0.0150 (6)	0.0170 (6)	0.0175 (6)	0.0001 (4)	−0.0012 (5)	0.0024 (4)
B2	0.0148 (6)	0.0190 (6)	0.0193 (6)	0.0010 (5)	−0.0016 (5)	−0.0020 (5)
C1	0.0296 (7)	0.0292 (7)	0.0313 (7)	0.0020 (6)	−0.0147 (6)	−0.0049 (6)
C2	0.0177 (6)	0.0207 (5)	0.0188 (5)	0.0035 (4)	−0.0051 (4)	0.0003 (4)
C3	0.0247 (7)	0.0314 (7)	0.0195 (6)	0.0033 (5)	0.0000 (5)	0.0052 (5)
C4	0.0174 (6)	0.0360 (7)	0.0341 (7)	−0.0024 (5)	−0.0016 (5)	0.0115 (6)
C5	0.0137 (5)	0.0219 (5)	0.0186 (5)	0.0025 (4)	−0.0029 (4)	0.0029 (4)
C6	0.0224 (6)	0.0256 (6)	0.0254 (6)	0.0085 (5)	−0.0020 (5)	0.0009 (5)
C7	0.0135 (5)	0.0174 (5)	0.0171 (5)	0.0020 (4)	−0.0002 (4)	0.0009 (4)
C8	0.0160 (5)	0.0173 (5)	0.0169 (5)	0.0024 (4)	0.0013 (4)	0.0002 (4)
C9	0.0151 (5)	0.0155 (5)	0.0148 (5)	0.0001 (4)	−0.0010 (4)	0.0005 (4)
C10	0.0124 (5)	0.0150 (5)	0.0146 (5)	−0.0003 (4)	−0.0006 (4)	0.0009 (4)
C11	0.0240 (6)	0.0213 (5)	0.0159 (5)	0.0017 (5)	−0.0047 (4)	−0.0016 (4)
C12	0.0218 (6)	0.0249 (6)	0.0263 (6)	0.0005 (5)	−0.0074 (5)	−0.0040 (5)
C13	0.0241 (7)	0.0259 (6)	0.0263 (6)	−0.0023 (5)	−0.0026 (5)	−0.0022 (5)
C14	0.0244 (7)	0.0275 (6)	0.0277 (7)	−0.0027 (5)	−0.0052 (5)	−0.0028 (5)
C15	0.0267 (7)	0.0267 (7)	0.0427 (8)	−0.0018 (6)	−0.0028 (6)	−0.0002 (6)
C16	0.0362 (9)	0.0270 (7)	0.0508 (10)	−0.0042 (6)	−0.0023 (7)	0.0037 (7)
C17	0.0125 (5)	0.0160 (5)	0.0143 (5)	−0.0002 (4)	0.0001 (4)	−0.0011 (4)
C18	0.0162 (5)	0.0164 (5)	0.0151 (5)	0.0016 (4)	0.0004 (4)	−0.0003 (4)
C19	0.0181 (6)	0.0167 (5)	0.0178 (5)	0.0040 (4)	−0.0015 (4)	−0.0003 (4)
C20	0.0151 (5)	0.0182 (5)	0.0185 (5)	0.0019 (4)	−0.0014 (4)	−0.0017 (4)
C21	0.0242 (6)	0.0175 (5)	0.0189 (5)	0.0016 (5)	0.0049 (5)	0.0016 (4)
C22	0.0424 (9)	0.0217 (6)	0.0232 (6)	−0.0055 (6)	0.0115 (6)	−0.0010 (5)
C23	0.0335 (8)	0.0196 (6)	0.0241 (6)	0.0004 (5)	0.0076 (5)	0.0039 (5)
C24	0.0293 (7)	0.0315 (7)	0.0248 (6)	0.0027 (6)	0.0009 (5)	0.0078 (5)
C25	0.0238 (7)	0.0341 (7)	0.0252 (7)	−0.0029 (5)	0.0022 (5)	0.0031 (5)
C26	0.0304 (8)	0.0345 (7)	0.0245 (7)	−0.0019 (6)	0.0052 (6)	−0.0020 (5)
C27	0.0176 (6)	0.0220 (6)	0.0291 (6)	0.0064 (5)	0.0063 (5)	0.0062 (5)
C28	0.0164 (6)	0.0199 (5)	0.0228 (6)	0.0041 (4)	0.0038 (4)	0.0009 (4)
C29	0.0375 (9)	0.0540 (10)	0.0284 (7)	0.0162 (8)	0.0117 (6)	0.0098 (7)
C30	0.0246 (7)	0.0231 (7)	0.0732 (12)	−0.0018 (6)	0.0112 (8)	0.0030 (7)
C31	0.0265 (7)	0.0380 (8)	0.0382 (8)	0.0115 (6)	−0.0042 (6)	0.0049 (6)
C32	0.0288 (8)	0.0230 (6)	0.0464 (9)	−0.0026 (6)	0.0106 (7)	−0.0076 (6)
O1	0.0206 (4)	0.0220 (4)	0.0202 (4)	0.0071 (3)	−0.0061 (3)	−0.0023 (3)
O2	0.0148 (4)	0.0229 (4)	0.0171 (4)	0.0036 (3)	−0.0029 (3)	0.0000 (3)
O3	0.0172 (4)	0.0299 (5)	0.0261 (5)	0.0092 (4)	0.0052 (4)	0.0081 (4)
O4	0.0223 (5)	0.0248 (4)	0.0193 (4)	0.0089 (4)	0.0043 (3)	0.0022 (3)
S1	0.01508 (14)	0.01852 (14)	0.01509 (13)	0.00182 (10)	−0.00279 (10)	−0.00195 (10)
S2	0.01368 (14)	0.01725 (13)	0.01810 (14)	0.00051 (10)	0.00189 (10)	0.00161 (10)

### Geometric parameters (Å, °)

**Table:**

B1—O2	1.3638 (16)	C16—H16A	0.9800
B1—O1	1.3715 (16)	C16—H16B	0.9800
B1—C7	1.5449 (18)	C16—H16C	0.9800
B2—O4	1.3654 (16)	C17—C18	1.3767 (16)
B2—O3	1.3660 (17)	C17—S2	1.7265 (12)
B2—C20	1.5408 (18)	C18—C19	1.4225 (16)
C1—C2	1.5181 (18)	C18—C21	1.5030 (17)
C1—H1A	0.9800	C19—C20	1.3715 (17)
C1—H1B	0.9800	C19—H19	0.9500
C1—H1C	0.9800	C20—S2	1.7218 (12)
C2—O1	1.4631 (14)	C21—C22	1.5233 (18)
C2—C3	1.5244 (19)	C21—H21A	0.9900
C2—C5	1.5627 (18)	C21—H21B	0.9900
C3—H3A	0.9800	C22—C23	1.5254 (19)
C3—H3B	0.9800	C22—H22A	0.9900
C3—H3C	0.9800	C22—H22B	0.9900
C4—C5	1.5223 (18)	C23—C24	1.520 (2)
C4—H4A	0.9800	C23—H23A	0.9900
C4—H4B	0.9800	C23—H23B	0.9900
C4—H4C	0.9800	C24—C25	1.5301 (19)
C5—O2	1.4681 (14)	C24—H24A	0.9900
C5—C6	1.5184 (18)	C24—H24B	0.9900
C6—H6A	0.9800	C25—C26	1.518 (2)
C6—H6B	0.9800	C25—H25A	0.9900
C6—H6C	0.9800	C25—H25B	0.9900
C7—C8	1.3785 (16)	C26—H26A	0.9800
C7—S1	1.7223 (12)	C26—H26B	0.9800
C8—C9	1.4199 (16)	C26—H26C	0.9800
C8—H8	0.9500	C27—O3	1.4654 (15)
C9—C10	1.3826 (16)	C27—C29	1.509 (2)
C9—C11	1.5086 (16)	C27—C30	1.526 (2)
C10—C17	1.4696 (16)	C27—C28	1.5587 (18)
C10—S1	1.7206 (12)	C28—O4	1.4598 (15)
C11—C12	1.5434 (19)	C28—C31	1.510 (2)
C11—H11A	0.9900	C28—C32	1.5196 (19)
C11—H11B	0.9900	C29—H29A	0.9800
C12—C13	1.5204 (19)	C29—H29B	0.9800
C12—H12A	0.9900	C29—H29C	0.9800
C12—H12B	0.9900	C30—H30A	0.9800
C13—C14	1.524 (2)	C30—H30B	0.9800
C13—H13A	0.9900	C30—H30C	0.9800
C13—H13B	0.9900	C31—H31A	0.9800
C14—C15	1.522 (2)	C31—H31B	0.9800
C14—H14A	0.9900	C31—H31C	0.9800
C14—H14B	0.9900	C32—H32A	0.9800
C15—C16	1.518 (2)	C32—H32B	0.9800
C15—H15A	0.9900	C32—H32C	0.9800
C15—H15B	0.9900		
			
O2—B1—O1	114.00 (11)	C18—C17—C10	128.00 (11)
O2—B1—C7	124.42 (11)	C18—C17—S2	111.58 (9)
O1—B1—C7	121.59 (11)	C10—C17—S2	120.36 (8)
O4—B2—O3	114.12 (11)	C17—C18—C19	111.02 (11)
O4—B2—C20	121.21 (11)	C17—C18—C21	125.73 (11)
O3—B2—C20	124.66 (11)	C19—C18—C21	123.22 (10)
C2—C1—H1A	109.5	C20—C19—C18	114.97 (11)
C2—C1—H1B	109.5	C20—C19—H19	122.5
H1A—C1—H1B	109.5	C18—C19—H19	122.5
C2—C1—H1C	109.5	C19—C20—B2	125.70 (11)
H1A—C1—H1C	109.5	C19—C20—S2	109.71 (9)
H1B—C1—H1C	109.5	B2—C20—S2	124.59 (9)
O1—C2—C1	108.66 (10)	C18—C21—C22	112.57 (10)
O1—C2—C3	106.41 (11)	C18—C21—H21A	109.1
C1—C2—C3	110.22 (11)	C22—C21—H21A	109.1
O1—C2—C5	102.00 (9)	C18—C21—H21B	109.1
C1—C2—C5	115.42 (12)	C22—C21—H21B	109.1
C3—C2—C5	113.32 (11)	H21A—C21—H21B	107.8
C2—C3—H3A	109.5	C21—C22—C23	113.81 (11)
C2—C3—H3B	109.5	C21—C22—H22A	108.8
H3A—C3—H3B	109.5	C23—C22—H22A	108.8
C2—C3—H3C	109.5	C21—C22—H22B	108.8
H3A—C3—H3C	109.5	C23—C22—H22B	108.8
H3B—C3—H3C	109.5	H22A—C22—H22B	107.7
C5—C4—H4A	109.5	C24—C23—C22	114.17 (12)
C5—C4—H4B	109.5	C24—C23—H23A	108.7
H4A—C4—H4B	109.5	C22—C23—H23A	108.7
C5—C4—H4C	109.5	C24—C23—H23B	108.7
H4A—C4—H4C	109.5	C22—C23—H23B	108.7
H4B—C4—H4C	109.5	H23A—C23—H23B	107.6
O2—C5—C6	108.64 (10)	C23—C24—C25	113.38 (12)
O2—C5—C4	106.51 (10)	C23—C24—H24A	108.9
C6—C5—C4	110.27 (11)	C25—C24—H24A	108.9
O2—C5—C2	102.49 (9)	C23—C24—H24B	108.9
C6—C5—C2	114.94 (10)	C25—C24—H24B	108.9
C4—C5—C2	113.27 (11)	H24A—C24—H24B	107.7
C5—C6—H6A	109.5	C26—C25—C24	113.00 (12)
C5—C6—H6B	109.5	C26—C25—H25A	109.0
H6A—C6—H6B	109.5	C24—C25—H25A	109.0
C5—C6—H6C	109.5	C26—C25—H25B	109.0
H6A—C6—H6C	109.5	C24—C25—H25B	109.0
H6B—C6—H6C	109.5	H25A—C25—H25B	107.8
C8—C7—B1	130.43 (11)	C25—C26—H26A	109.5
C8—C7—S1	109.62 (9)	C25—C26—H26B	109.5
B1—C7—S1	119.94 (9)	H26A—C26—H26B	109.5
C7—C8—C9	114.77 (11)	C25—C26—H26C	109.5
C7—C8—H8	122.6	H26A—C26—H26C	109.5
C9—C8—H8	122.6	H26B—C26—H26C	109.5
C10—C9—C8	111.10 (10)	O3—C27—C29	109.17 (12)
C10—C9—C11	123.51 (11)	O3—C27—C30	105.59 (11)
C8—C9—C11	125.30 (11)	C29—C27—C30	110.59 (14)
C9—C10—C17	128.56 (10)	O3—C27—C28	103.03 (10)
C9—C10—S1	111.59 (9)	C29—C27—C28	115.76 (12)
C17—C10—S1	119.84 (8)	C30—C27—C28	111.90 (12)
C9—C11—C12	111.84 (10)	O4—C28—C31	108.74 (11)
C9—C11—H11A	109.2	O4—C28—C32	106.37 (11)
C12—C11—H11A	109.2	C31—C28—C32	110.03 (12)
C9—C11—H11B	109.2	O4—C28—C27	102.50 (9)
C12—C11—H11B	109.2	C31—C28—C27	115.23 (12)
H11A—C11—H11B	107.9	C32—C28—C27	113.25 (12)
C13—C12—C11	113.99 (11)	C27—C29—H29A	109.5
C13—C12—H12A	108.8	C27—C29—H29B	109.5
C11—C12—H12A	108.8	H29A—C29—H29B	109.5
C13—C12—H12B	108.8	C27—C29—H29C	109.5
C11—C12—H12B	108.8	H29A—C29—H29C	109.5
H12A—C12—H12B	107.6	H29B—C29—H29C	109.5
C12—C13—C14	113.64 (12)	C27—C30—H30A	109.5
C12—C13—H13A	108.8	C27—C30—H30B	109.5
C14—C13—H13A	108.8	H30A—C30—H30B	109.5
C12—C13—H13B	108.8	C27—C30—H30C	109.5
C14—C13—H13B	108.8	H30A—C30—H30C	109.5
H13A—C13—H13B	107.7	H30B—C30—H30C	109.5
C15—C14—C13	113.96 (13)	C28—C31—H31A	109.5
C15—C14—H14A	108.8	C28—C31—H31B	109.5
C13—C14—H14A	108.8	H31A—C31—H31B	109.5
C15—C14—H14B	108.8	C28—C31—H31C	109.5
C13—C14—H14B	108.8	H31A—C31—H31C	109.5
H14A—C14—H14B	107.7	H31B—C31—H31C	109.5
C16—C15—C14	113.33 (14)	C28—C32—H32A	109.5
C16—C15—H15A	108.9	C28—C32—H32B	109.5
C14—C15—H15A	108.9	H32A—C32—H32B	109.5
C16—C15—H15B	108.9	C28—C32—H32C	109.5
C14—C15—H15B	108.9	H32A—C32—H32C	109.5
H15A—C15—H15B	107.7	H32B—C32—H32C	109.5
C15—C16—H16A	109.5	B1—O1—C2	106.83 (9)
C15—C16—H16B	109.5	B1—O2—C5	106.36 (9)
H16A—C16—H16B	109.5	B2—O3—C27	106.50 (10)
C15—C16—H16C	109.5	B2—O4—C28	107.21 (10)
H16A—C16—H16C	109.5	C10—S1—C7	92.91 (6)
H16B—C16—H16C	109.5	C20—S2—C17	92.72 (6)
			
O1—C2—C5—O2	−28.30 (11)	C17—C18—C21—C22	−121.33 (14)
C1—C2—C5—O2	−145.90 (11)	C19—C18—C21—C22	56.35 (16)
C3—C2—C5—O2	85.67 (12)	C18—C21—C22—C23	−167.13 (12)
O1—C2—C5—C6	−145.95 (11)	C21—C22—C23—C24	−66.75 (18)
C1—C2—C5—C6	96.45 (14)	C22—C23—C24—C25	−177.15 (12)
C3—C2—C5—C6	−31.98 (15)	C23—C24—C25—C26	−71.53 (17)
O1—C2—C5—C4	86.04 (12)	O3—C27—C28—O4	25.36 (13)
C1—C2—C5—C4	−31.56 (15)	C29—C27—C28—O4	144.44 (12)
C3—C2—C5—C4	−159.99 (11)	C30—C27—C28—O4	−87.62 (13)
O2—B1—C7—C8	3.4 (2)	O3—C27—C28—C31	143.28 (12)
O1—B1—C7—C8	−176.39 (12)	C29—C27—C28—C31	−97.64 (16)
O2—B1—C7—S1	−177.69 (10)	C30—C27—C28—C31	30.30 (17)
O1—B1—C7—S1	2.55 (17)	O3—C27—C28—C32	−88.81 (13)
B1—C7—C8—C9	178.53 (12)	C29—C27—C28—C32	30.27 (17)
S1—C7—C8—C9	−0.50 (14)	C30—C27—C28—C32	158.21 (12)
C7—C8—C9—C10	0.85 (15)	O2—B1—O1—C2	−9.18 (14)
C7—C8—C9—C11	−175.73 (11)	C7—B1—O1—C2	170.61 (11)
C8—C9—C10—C17	177.90 (11)	C1—C2—O1—B1	145.43 (12)
C11—C9—C10—C17	−5.45 (19)	C3—C2—O1—B1	−95.90 (12)
C8—C9—C10—S1	−0.80 (13)	C5—C2—O1—B1	23.08 (12)
C11—C9—C10—S1	175.85 (9)	O1—B1—O2—C5	−10.37 (14)
C10—C9—C11—C12	−77.10 (15)	C7—B1—O2—C5	169.85 (11)
C8—C9—C11—C12	99.07 (14)	C6—C5—O2—B1	145.86 (11)
C9—C11—C12—C13	−72.44 (14)	C4—C5—O2—B1	−95.37 (12)
C11—C12—C13—C14	169.15 (11)	C2—C5—O2—B1	23.82 (12)
C12—C13—C14—C15	165.11 (12)	O4—B2—O3—C27	9.09 (15)
C13—C14—C15—C16	176.88 (13)	C20—B2—O3—C27	−172.44 (12)
C9—C10—C17—C18	−68.23 (18)	C29—C27—O3—B2	−144.77 (13)
S1—C10—C17—C18	110.38 (13)	C30—C27—O3—B2	96.31 (13)
C9—C10—C17—S2	114.69 (12)	C28—C27—O3—B2	−21.22 (13)
S1—C10—C17—S2	−66.71 (12)	O3—B2—O4—C28	8.41 (15)
C10—C17—C18—C19	−177.50 (11)	C20—B2—O4—C28	−170.13 (11)
S2—C17—C18—C19	−0.21 (13)	C31—C28—O4—B2	−143.23 (12)
C10—C17—C18—C21	0.4 (2)	C32—C28—O4—B2	98.31 (13)
S2—C17—C18—C21	177.72 (10)	C27—C28—O4—B2	−20.80 (13)
C17—C18—C19—C20	0.33 (15)	C9—C10—S1—C7	0.46 (9)
C21—C18—C19—C20	−177.65 (11)	C17—C10—S1—C7	−178.37 (10)
C18—C19—C20—B2	179.11 (11)	C8—C7—S1—C10	0.02 (10)
C18—C19—C20—S2	−0.30 (14)	B1—C7—S1—C10	−179.12 (10)
O4—B2—C20—C19	15.00 (19)	C19—C20—S2—C17	0.15 (10)
O3—B2—C20—C19	−163.37 (13)	B2—C20—S2—C17	−179.27 (11)
O4—B2—C20—S2	−165.68 (10)	C18—C17—S2—C20	0.04 (9)
O3—B2—C20—S2	15.95 (18)	C10—C17—S2—C20	177.57 (10)

### Hydrogen-bond geometry (Å, °)

**Table:**

*D*—H···*A*	*D*—H	H···*A*	*D*···*A*	*D*—H···*A*
C30—H30C···O1^i^	0.98	2.53	3.2984 (18)	136.

Symmetry codes: (i) *x*−1, *y*, *z*.
